# Bridging human and machine intelligence: Reverse-engineering radiologist intentions for clinical trust and adoption

**DOI:** 10.1016/j.csbj.2024.11.012

**Published:** 2024-11-08

**Authors:** Akash Awasthi, Ngan Le, Zhigang Deng, Rishi Agrawal, Carol C. Wu, Hien Van Nguyen

**Affiliations:** a Department of Electrical and Computer Engineering, University of Houston, United States; b Department of Computer Science & Computer Engineering, University of Arkansas, United States; c Department of Computer Science, University of Houston, Houston, TX, United States; d Department of Thoracic Imaging, Division of Diagnostic Imaging, The University of Texas MD Anderson Cancer Center, Houston, TX, United States

**Keywords:** DVC(Deep video captioning), TGID, Intention, LMM(Large Multimodal Model)

## Abstract

In the rapidly evolving landscape of medical imaging, the integration of artificial intelligence (AI) with clinical expertise offers unprecedented opportunities to enhance diagnostic precision and accuracy. Yet, the "black box" nature of AI models often limits their integration into clinical practice, where transparency and interpretability are important. This paper presents a novel system leveraging the Large Multimodal Model (LMM) to bridge the gap between AI predictions and the cognitive processes of radiologists. This system consists of two core modules, Temporally Grounded Intention Detection (TGID) and Region Extraction (RE). The TGID module predicts the radiologist's intentions by analyzing eye gaze fixation heatmap videos and corresponding radiology reports. Additionally, the RE module extracts regions of interest that align with these intentions, mirroring the radiologist’s diagnostic focus. This approach introduces a new task, radiologist intention detection, and is the first application of Dense Video Captioning (DVC) in the medical domain. By making AI systems more interpretable and aligned with radiologist’s cognitive processes, this proposed system aims to enhance trust, improve diagnostic accuracy, and support medical education. Additionally, it holds the potential for automated error correction, guiding junior radiologists, and fostering more effective training and feedback mechanisms. This work sets a precedent for future research in AI-driven healthcare, offering a pathway towards transparent, trustworthy, and human-centered AI systems. We evaluated this model using NLG(Natural Language Generation), time-related, and vision-based metrics, demonstrating superior performance in generating temporally grounded intentions on REFLACX and EGD-CXR datasets. This model also demonstrated strong predictive accuracy in overlap scores for medical abnormalities and effective region extraction with high IoU(Intersection over Union), especially in complex cases like cardiomegaly and edema. These results highlight the system's potential to enhance diagnostic accuracy and support continuous learning in radiology.

## Graphical Abstract

**Figure:**
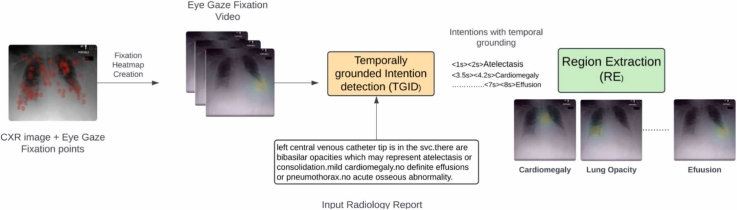
Overview of our proposed system, comprising two key submodules: Temporally Grounded Intention Detection (TGID) and Region Extraction (RE). The system processes eye gaze fixation video overlaid on CXR images alongside the corresponding radiology report, ultimately identifying the intended diagnosis and highlighting the associated Regions of Interest (ROI).

## 1 Introduction

In recent years, the integration of artificial intelligence (AI) into medical imaging has led to significant advancements, offering the potential to enhance diagnostic accuracy and streamline clinical workflows [1], [2]. However, alongside these developments, a critical challenge is to ensure that AI systems are not only powerful in their predictive capabilities but also transparent and interpretable [3], [4], [5]. In clinical practice, interpretability is essential for building trust and enabling radiologists to rely on AI in making high-stakes decisions [6], [7], [8], [9]. Without this, the adoption of AI systems in healthcare remains limited [10].

Radiologists, as expert interpreters of medical images, do not solely rely on isolated image features or patterns detected by machines. Instead, they bring to bear a combination of clinical knowledge, years of experience, and an intuitive, intention-driven process for interpreting complex medical data [11], [12]. They focus on specific regions of an image to investigate abnormalities, confirm suspicions, or rule out certain conditions. This decision-making process is deeply rooted in their reasoning and cognitive strategies, which are difficult to replicate or explain through traditional AI models [12], [13].

Traditional AI systems, while effective at recognizing patterns, are typically data-driven and lack insight into human cognitive processes [14]. These systems generate predictions without providing an explanation that aligns with how a radiologist might interpret the same image [15]. This "black-box" nature of AI creates a significant disconnect between machine predictions and human understanding, resulting in skepticism and eroding trust among medical professionals [16]. As a result, even when AI systems produce accurate predictions, they are also prone to errors such as false positives and false negatives. In particular, false positive findings can waste a radiologist's time if no explanation is provided, further complicating reliance on AI for critical diagnostic decisions.

For AI to be truly integrated into clinical practice, it must offer more than just accurate predictions. It must provide transparency and explainability in a way that corresponds to the radiologist’s thought process and workflow [17]. Addressing this gap between AI outputs and human cognitive processes is essential for fostering trust and ensuring the successful adoption of AI in healthcare.

Eye gaze data provides a promising solution to this challenge. By capturing where radiologists focus their attention when interpreting chest X-ray (CXR) images [18], [19], [20]. It reveals the regions of interest that radiologists prioritize during diagnosis, providing valuable insight into their reasoning and intentions [21]. Leveraging this rich source of information, we propose a novel approach that uses eye gaze data to enhance the interpretability of AI systems in medical imaging.

**Fig. 1:**
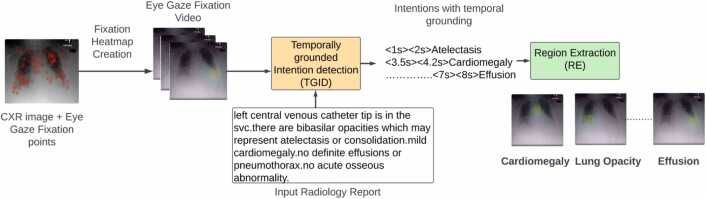
Overview of our proposed system, comprising two key submodules: Temporally Grounded Intention Detection (TGID) and Region Extraction (RE). The system processes eye gaze fixation video overlaid on CXR images alongside the corresponding radiology report, ultimately identifying the intended diagnosis and highlighting the associated Regions of Interest (ROI).

This paper introduces a novel system based on the Large Multimodal Models ( LMM) designed to bridge this gap by aligning AI outputs with the cognitive processes of radiologists and adding more explainability in radiological diagnosis. The proposed system comprehends and predicts the intentions of radiologists as they interpret medical images, effectively creating a synergy between AI and human expertise. As shown in Fig. 1, The system comprises two key modules: Temporally Grounded Intention Detection (TGID) and Region Extraction (RE). The TGID module analyzes fixation heatmap videos and corresponding radiology reports to predict the intentions driving specific gaze patterns, providing a temporal grounding for these intentions. The RE module utilizes these predictions to extract relevant regions of interest, offering a visual representation that mirrors the radiologist's focus during the diagnostic process.

The development of this system marks the introduction of a new task within the medical domain—radiologist intention detection. To ensure the practical relevance and accuracy of our approach, we have established comprehensive evaluation strategies to assess the performance of the system. Notably, this work represents the first attempt to apply Dense Video Captioning (DVC) [22], [23], [24], [25], [26] to medical data, setting a precedent for future research in AI-driven healthcare.

The applications of this system extend far beyond simply enhancing diagnostic tools. By making AI more interpretable and aligned with human cognition, this approach can significantly improve trust among medical professionals, thereby increasing the likelihood of AI adoption in clinical settings. This, in turn, could lead to more efficient workflows and better patient outcomes. The system also has substantial potential in medical education, offering trainees deep insights into expert diagnostic processes. It can help trainee radiologists understand how experienced radiologists make decisions about multiple abnormalities and identify the regions of interest corresponding to each intention mentioned in radiology reports—essentially reverse engineering the radiologist’s intentions. Moreover, it could serve as a vital component in automated error correction systems [27], rectifying perceptual errors. The system can be leveraged to guide trainees towards identifying the correct regions of interest and to enhance training and feedback.

The key contributions of this paper include:

• Development of a novel system for comprehending the intentions of radiologists alongside the corresponding regions of interest.
• Introduction of evaluation strategies for assessing the performance and practical relevance of the proposed model.
• Pioneering a new task known as radiologist intention detection within the medical domain.
• Application of DVC to medical data, representing the first such attempt in this field.

## 2 Related work

The integration of AI in medical imaging has garnered significant attention in recent years, particularly in enhancing diagnostic accuracy and facilitating clinical workflows [28], [29], [30]. Several studies have explored AI applications in the interpretation of CXR images, focusing on different aspects such as abnormality detection [31], lesion localization [32], and disease classification [33]. These efforts have demonstrated the potential of AI to augment the capabilities of radiologists, reduce workload, and improve diagnostic efficiency. However, challenges remain in aligning AI predictions with the cognitive processes of human experts to ensure trust and interpretability in clinical practice.

a. **Interpretability and trust in AI systems**
  The interpretability of AI models in medical imaging has garnered significant attention, as it is essential for ensuring that healthcare practitioners can trust and effectively utilize these systems in clinical practice [6], [7], [8], [9]. Given the high stakes involved in medical decisions, understanding how AI systems arrive at their conclusions enhances user confidence, facilitates collaboration, and ultimately improves patient outcomes. Various approaches have been proposed to enhance the transparency of model predictions [34], [35], [36]. Explainable AI (XAI) techniques [37], [38], including saliency maps [39], Grad-CAM [40], and LIME [39], are widely utilized to illuminate the features that influence model decisions. New methods such as counterfactual explanations and causal reasoning have emerged to provide actionable insights (Kim et al., 2022) [41]. Additionally, Rudin et al. [42] advocate for inherently interpretable models, which eliminate the need for post-hoc explanations and foster greater user trust.
  Innovations in interpretability continue to evolve, with approaches such as prototype-based explanations [43] and enhanced integrated gradients [44]. The adoption of Vision Transformers for attention-based interpretability is also gaining traction [45], [46]. Furthermore, SHAP-based approaches [47], [48] provide valuable case-specific insights, while self-supervised learning [49] and Bayesian deep learning [50] further enhance interpretability by improving model understanding. Multi-modal AI [51] and interactive machine learning [52] are also pushing the boundaries of AI interpretability, striving to align models more closely with clinical reasoning and fostering greater trust in AI systems.
  Despite these advancements, existing methods primarily focus on elucidating the inner workings of AI models. However, they often fail to align with the nuanced thought processes of radiologists, who interpret medical images through a combination of clinical knowledge and visual inspection. This disconnect between AI outputs and human cognitive processes can hinder the successful integration of AI into clinical workflows, where trust and reliability are critical.
b. **Understanding radiologist’s cognitive processes and eye gaze:**
  Understanding the cognitive processes of radiologists during image interpretation is crucial for developing AI systems that complement human expertise [53]. Eye-tracking technology has been extensively used to study radiologist’s gaze patterns to gain insights into their diagnostic reasoning and decision-making strategies. Drew et al. [54] demonstrated that radiologist’s eye movements reflect their experience level, as expert radiologists tend to have more focused and efficient gaze patterns when identifying abnormalities. Similarly, [55] found that expert radiologists spend less time fixating on non-diagnostic areas, suggesting a more refined search strategy compared to novices.
  Recent studies have explored the application of eye-tracking data to develop models that mimic radiologist’s visual attention patterns. For example, Peng et al. [56] used the eye gaze data to predict the radiology report. Bertram et al. [57] also used eye-tracking data to understand the diagnostic process of radiologists when interpreting mammograms, finding that gaze patterns could be predictive of diagnostic success, and highlighting the importance of aligning AI systems with human visual strategies.
c. **The role of eye gaze in enhancing AI interpretability:**
  The integration of eye-tracking data into AI models has been shown to improve the interpretability and trustworthiness of these systems [58]. By aligning AI predictions with the regions of interest identified by radiologists' gaze patterns, researchers aim to create more intuitive and user-friendly diagnostic tools. Additionally, the use of eye-tracking in high-stakes decision environments, such as clinical settings, allows for a granular assessment of how Explainable AI (XAI) influences physician decision-making. Recent studies have demonstrated that eye-tracking can reveal how different types of XAI explanations affect clinician behavior and attention, potentially improving the interaction between human experts and AI [59]
d. **Video-based analysis and dense video captioning (DVC):**

Video-based analysis has emerged as a promising approach to capturing the temporal dynamics of radiologist’s visual attention during image interpretation. DVC has been employed in various domains to generate temporally grounded descriptions of events in videos [22], [23], [24], [25], [26]. While DVC has not been extensively applied to medical imaging, its potential to provide a temporal context for interpreting gaze patterns and corresponding clinical intentions is significant. Our work represents the first attempt to apply DVC to the medical domain, specifically in understanding the temporally grounded intentions of radiologists during CXR interpretation.

Our proposed system builds upon these foundational studies by introducing a novel approach to understanding and modeling radiologists' intentions in CXR image analysis. TGID module and RE modules are designed to predict radiologists' cognitive processes and highlight relevant regions of interest, thus bridging the gap between AI predictions and human interpretation. This approach not only enhances the interpretability of AI models but also provides a valuable tool for error correction and medical education, particularly in guiding inexperienced practitioners.

## 3 Methodology

**Fig. 2:**
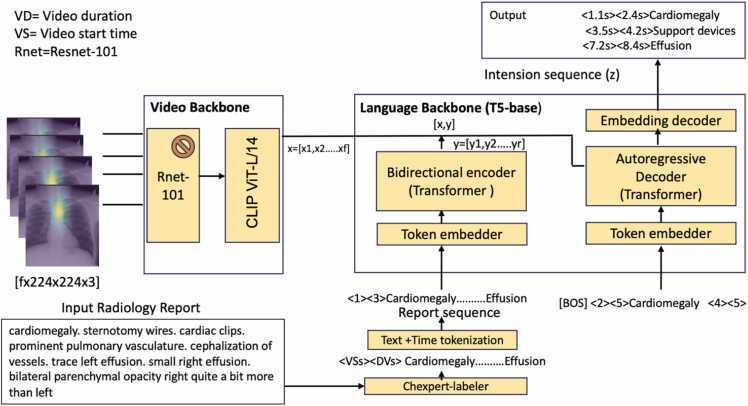
TGID module overview: A Large Multimodal Model (LMM) that takes video features and summarized radiology reports with appended time tokens as input and output the intention sequence with temporal grounding.

Our proposed system comprises two primary modules: 1) Temporally Grounded Intention Detection (TGID) and 2) Region Extraction (RE). The TGID module is based on the LMM which works on the multimodal data. Illustrated in Fig. 2, the TGID module utilizes the fixation heatmap video and the time steps embedded in the radiology report as inputs. It then predicts the main intentions in the radiology report with the corresponding temporal grounding or time steps.

**Fig. 3:**
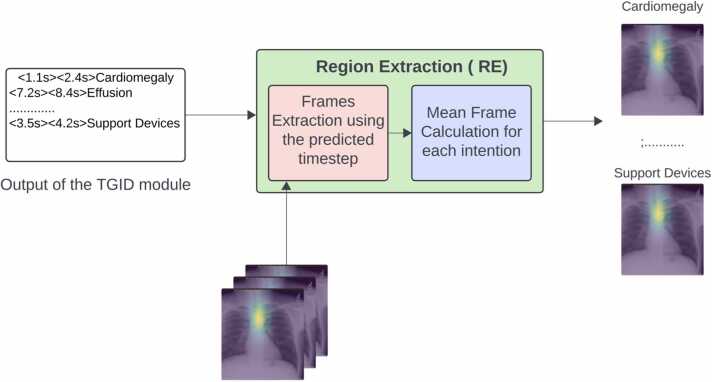
Region Extraction (RE) module overview: The RE module utilizes the predicted time steps (start and end times) and the identified intention to extract clips from the input video, containing multiple frames. Subsequently, we compute the mean of all images within the extracted clip to determine a representative image for the region of interest associated with the intended purpose.

As shown in Fig. 3, The RE module utilizes the predicted time steps (start and end times) and the identified intention to extract clips from the input video, containing multiple frames. Subsequently, we compute the mean of all images within the extracted clip to determine a representative image for the region of interest associated with the intention. It's important to note that the RE module is a straightforward search algorithm reliant on TGID predictions and is not the primary focus of our contribution.

The TGID module serves as the core of our system by predicting temporally grounded intentions. The intricate design of this module is depicted in Fig. 2. As illustrated, the proposed architecture comprises two integral components: the Video Backbone and the Language Backbone. We employed a Chexpert labeler [60] to condense the radiology report into the main Chexpert labels [60]. We call these labels intentions and for this study, we only focus on the seven intentions Cardiomegaly, Edema, Atelectasis, Lung Opacity, Pleural Effusion, and Pneumothorax. Chexpert-labeler produces the summarized radiology report as shown in Fig. 2. To enhance the summarized report, we added the start and end times, with the end time representing the video duration and the start time set at 1.1 s. This choice is grounded in our observation that radiologists typically commence speaking after 1.1 s upon viewing the video, a value derived from the analysis of the EGD-CXR [61] and REFLACX [62] datasets.

a. Video Backbone
  The Video Backbone plays a pivotal role in extracting features from the input video. It comprises a spatial encoder followed by a temporal encoder, operating on a sequence of 'f' frames. Utilizing a pre-trained Resnet-101 [63] as the spatial encoder, we extract individual frame features, considering the spatial characteristics of each frame in the video. The input set consists of videos with dimensions 'f × h × w × c,' where 'h,' 'w,' and 'c' represent the height, width, and number of channels of each frame. The spatial encoder processes each frame independently, and we maintain the spatial backbone as frozen to minimize computational costs and parameter count in the overall model.
  The spatial encoder generates a two-dimensional array, with the first dimension representing the number of frames and the second representing the embedding dimension. Although each video may have a varying number of frames, we limit our consideration to the features of the first 100 frames. To accommodate videos with fewer than 100 frames, we pad the feature extraction output from Resnet with zeros.
  For the temporal encoder, we employ a trained CLIP ViT-L/14 [64], [65] transformer to produce contextualized embeddings, contributing to the comprehensive feature representation of the input video.
b. **Language backbone**
  Our language backbone [22] is built on the Large Language Model( LLM) T5 [65], employing an encoder-decoder architecture. We initialized both the text encoder and decoder with the t5-base model, which underwent pretraining on web text corpora with a denoising loss.
c. **Text and time tokenization**
  We utilize the SentencePiece tokenizer [27] with a vocabulary size of V=32,128. Our approach involves initial text tokenization, and to augment this process, we incorporate two extra time tokens, bringing the total to V + 2 tokens. Throughout the training, these time tokens represent the initiation and conclusion times when the radiologist begins and concludes the depiction of the radiology report while examining the CXR image on the screen. The time tokenization process adheres to the equation detailed below.
  (1) $$\mathrm{tt}=\left|\frac{\left(\mathrm{ts}\times \;N\right)}{D}\right|$$
  In Eq. 1, "tt" denotes the time token, "ts" represents the timestep (indicating the start or end time step), "N" signifies the quantized bin with a specified value of 100 (N = bins), and "D" corresponds to the video duration.
d. **Text encoder**
  It accepts a report sequence as input, where the report sequence comprises 'r' tokens denoted as 'y' belonging to the set $y\in {\left\{1,\;...,V+N\right\}}^{r}\;$. Here, 'v' represents the vocabulary size of the text, 'n' is the size of time tokens (here n=2), and 'r' stands for the total number of tokens in the report sequence. The text encoder includes an embedding layer responsible for independently embedding each token, producing a semantic embedding of size 'rxd'. Subsequently, a transformer encoder calculates contextualized embeddings of size 'rxd', with 'd' representing the hidden dimension.
e. **Text decoder**
  Comprising a transformer decoder and an embedding layer, the system generates an intention sequence with associated temporal grounding, referred to as the intention sequence. Each abnormality k is characterized by a text segment, a start time, and an end time. We first construct for each event k a sequence by concatenating its start time token t(startk), its end time token t(end), and its text tokens $\left[z_{k1},\;...\;,\;z_{kl_{k}}\right]$. Finally, the event sequence is obtained by prepending and appending a BOS and EOS tokens to indicate the start and the end of the sequence, respectively, i.e. $z=\;[\mathrm{BOS},\;t_{\mathrm{start}1}\;,\;t_{\mathrm{end}1\;},\;z_{1_{1}}\;,\;...\;,\;z_{1_{l_{1}}}\;,\;t_{\mathrm{start}2\;}\;,\;...\;,\mathrm{EOS}].$
  The transformer decoder, functioning causally, employs cross-attention with the encoder output, formed by concatenating visual and encoder transformer embeddings (x_t and y_t), along with all tokens generated earlier. Simultaneously, it performs self-attention across the entire set of previously generated tokens. The text decoder produces the event sequence z by utilizing an embedding decoder, which is applied on top of the transformer text decoder. This decoder predicts the probability distribution over the joint vocabulary of text and time tokens, enabling the model to anticipate the subsequent token in the report sequence.
f. **Pretraining & finetuning**

We utilized the pre-trained model from vid2seq [22], specifically trained on the ActivityNet Captions dataset [66], which comprises approximately 20,000 untrimmed videos depicting diverse human activities. Each video is accompanied by transcribed speech sentences and timestamps, establishing a temporal connection to events. Given the limited availability of fixation videos and corresponding transcriptions in the medical domain, leveraging this pre-trained model enables our system to understand long-term relationships among different speech segments.

Subsequently, during the finetuning stage, the model is refined to predict the intention sequence (intention + time interval) by considering both the summarized radiology report sequence obtained through the CheXpert labeler and the visual sequence. The fine-tuning objective is derived from the maximum likelihood objective, elaborated upon in this context [22]. The primary goal of this TGID module is to understand human cognition during decision-making in abnormal diagnosis. In simpler terms, this module learns what radiologists focus on when making decisions based on CXR images.

## 4 Datasets & experimentation

For this study, we selected the EGD-CXR [61] and REFLACX [62] datasets due to the limited availability of eye-tracking datasets specifically focused on radiology and medical imaging. At the time of our research, these were the only two datasets that combined both eye-tracking data and CXR images, making them essential resources for our investigation into radiologist intention prediction. EGD-CXR dataset comprises 1071 CXR images reviewed by a radiologist using an eye-tracking system. Meanwhile, the REFLACX dataset encompasses 2344 cases with synchronized eye-tracking and transcription pairs, annotated by five radiologists. We utilized two different datasets recorded by different radiologists with varying levels of experience. EGD-CXR is recorded on a single experienced radiologist but the REFLACX is recorded on the 5 different radiologists with varying levels of experience. We have trained and tested our model on EGD-CXR and REFLACX respectively. A detailed description of the training and test datasets of EGD-CXR and REFLACX is provided in Table 1.

a) **Dataset preprocessing:**

**Table 1:** Overview of training and test data allocation from EGD-CXR and REFLACX datasets.

**Dataset**	**Training set (∼75%)**	**Test set (∼25%)**
EGD-CXR	800	271
REFLACX	1772	572

**Fig. 4:**
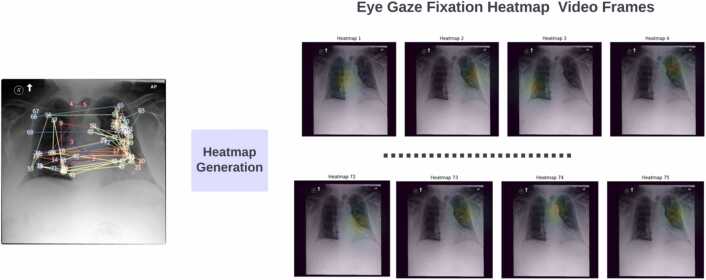
Demonstration of Creating the eye gaze heatmap videos which contain the frames representing the overlaid eye gaze fixation heatmap on the actual CXR image.

Our proposed system takes eye-gaze heatmap videos overlaid on the corresponding CXR images as input. We created the eye gaze heatmap videos by overlaying the eye gaze fixation heatmap on the original CXR image. However, the eye gaze fixation heatmaps are created using the fixation points provided in the eye gaze data and Gaussian intensity is used to create the intensity around the fixation points using the sigma= 150. We used the same procedure to create the heatmaps as used in this work [61]. They have provided the code in their GitHub repository to create the heatmaps using fixations points. A few instances of the heatmap video from the REFLACX data are shown in Fig. 4. The frame rate from these videos is 1. Input to the proposed system is the radiology report and the eye gaze video overlaid on the image.

To train the TGIP module, we used processed eye gaze videos overlaid on the CXR image and ground truth file which we created using the radiologist transcription which is provided in each eye gaze dataset ( EGD-CXR and REFLACX).

In our preprocessing phase, we summarize the real radiology reports to acquire both the ground truth and input report necessary for training the TGID module. When condensing the radiology reports, we prioritize essential abnormalities outlined in the CheXpert labeler. Our approach of preprocessing aims to avoid converting the entire radiology report into isolated labels. Instead, we focus on ensuring that the model grasps the fundamental aspects of chest X-ray anatomy, including spatial relations like "right" and "left" lungs. Radiology reports typically consist of multiple sentences separated by periods. To address this structure, our preprocessing methodology involves extracting each sentence. During this extraction process, we meticulously scrutinize each sentence for phrases corresponding to abnormalities in the CheXpert labeler. If a match is found, we substitute the sentence with the relevant abnormality; otherwise, it remains unchanged.

We extract the timestamps for each abnormality or unchanged sentence in the summarized report from the speech transcription associated with the CXR image. The speech transcription provides word-level timestamps. For each sentence in the actual report, we extract the start and end timestamps from the transcription and apply them to the corresponding sentence or label in the summarized report. This entire process, including report summarization and timestamp extraction, is detailed in Fig. 1 of the supplementary section. The dataset and code for this preprocessing step are also available in our repository. This resulting file served as the ground truth during the model fine-tuning, encompassing fixation heatmap videos and summarized radiology reports featuring only start and end timestamps for the entire report as input. This ensured a robust foundation for training. The core objective of the TGID module is to comprehend human cognition by predicting the timestamps associated with each intention. It's worth noting that our focus does not extend to conducting dense video captioning in this context.

It is important to recognize that there may be slight misalignment between eye gaze fixations and speech transcriptions. According to the REFLCAX dataset paper [62], the relationship between dictation and eye gaze fixation shows minimal delay; however, a correlation exists between expert annotations, represented as ellipses, and gaze fixation points [62]. This correlation suggests that eye gaze fixations can be utilized to extract the localization of abnormalities. The presence of this discernible signal in the data allows our model to derive valuable insights, even amidst the noise introduced by any temporal misalignment. To address the challenges associated with this misalignment, we integrated the capabilities of the TGID module, which is based on Google’s Vid2Seq model [22]. This model is specifically designed to handle instances of misalignment, making it particularly suitable for our application. The Vid2Seq model excels in learning from noisy supervision and is adept at jointly modeling narrations and timestamps. The original authors acknowledge that speech may not always accurately reflect the content of the visual data; nevertheless, the model successfully extracts meaningful relationships between the transcriptions and the visual data streams.

b) **Experimentation**

We used Python 3.8 for all the experiments. For training the machine learning model, we have used Python packages like Pytorch 1.13.1, Opencv-python 4.5.1.48, Numpy 1.22.4 Pandas 2.2.2, etc. For finetuning, we fine-tuned the TGID module on both the datasets REFLACX and EGD-CXR datasets separately. The model was trained on these datasets, consisting of fixation heatmap videos, summarized radiology reports, and temporally grounded intention sequences as ground truth. We employed Adam as the optimizer, with a batch size of 8 for both validation and training. We used 8 NVIDIA GPUS for finetuning the model on both the datasets. Notably, despite planning for 270 epochs of finetuning, the model demonstrated effective learning within approximately 50 epochs.

## 5 Evaluation metrics

In evaluating the performance of our system, we employ both time-based and vision-based metrics, specifically designed to assess how well the model predicts the temporal groundings and spatial regions of interest (ROI) for each intention (abnormality). These metrics ensure that the system aligns with the diagnostic workflow of radiologists, providing insights into both timing and visual accuracy.

a. Time-based metric for Intentions:
  To evaluate how accurately the model predicts the time intervals associated with each intention, we focus on measuring the overlap between the true and predicted time intervals in the intention sequence. Our goal is to assess how well the model temporally grounds each intention using gaze data (fixation videos).
  For each intention (abnormality), the model generates predictions defining specific time intervals. We calculate the overlap between the true and predicted intervals using the following formula:
  (2) $$\;\mathrm{Time Overlap score}=\;\frac{\mathrm{Length of the Overlapping Interval}}{\mathrm{Length of the True Interval}}$$
  In Eq. 2, the length of the Overlapping Interval is given by:
  Length of the Overlapping Interval=max(0,min(B2,B1)−max(A2,A1))
  The length of the True Interval is calculated as:
  Length of the True Interval=B1−A1
  Here, [A1, B1] represents the start and end times of the true interval, and [A2, B2] represents the start and end times of the predicted interval.
  The time-overlap score ranges from 0 to 1, where 0 signifies no overlap between the true and predicted intervals. By multiplying this score by 100, we obtain the percentage of overlap between these intervals, providing a clear measure of how well the model aligns with the true intervals.
b. Vision-Based metric for Intentions:

To evaluate the model’s spatial predictions, we use the Intersection over Union (IoU) metric, which measures the overlap between the true and predicted regions of interest for each intention. The IoU is a commonly used segmentation metric, also known as the **Jaccard Index**, and is calculated as follows:

(3) $$\;\mathrm{IoU}=\frac{\mathrm{Intersection}(\;A\cap B)}{\mathrm{Union}(A\cup B)}$$

In Eq. 3, intersection (A∩B) consists of pixels that are common between the prediction mask and the ground truth mask, while the union (A∪B) represents all pixels in either the prediction or ground truth mask. The IoU provides a measure of how closely the predicted regions align with the actual regions that the radiologist focused on during the diagnostic process.

For each intention, we first use the predicted start and end time steps to extract frames from the video. The mean image of these frames is then computed, representing the overall intensity in the predicted region of interest. Similarly, frames are extracted based on the ground truth time steps, and their mean image is calculated. Finally, we compute the IoU between the predicted and ground truth mean images, offering a quantitative assessment of the model's spatial alignment with the radiologist’s actual focus areas.

These metrics together offer a comprehensive evaluation of both the temporal and spatial aspects of the model's predictions.

## 6 Results & discussion

We begin by visualizing how our system predicts intentions (abnormalities) and diagnoses to understand the final output of the system. Following this, we evaluate the system using various metrics. To assess the proposed model's text generation capabilities, we employ natural language generation (NLG) metrics, comparing our results with state-of-the-art methods. Additionally, we conduct a thorough evaluation of the model's performance using time-related and vision-based metrics.

a. **Radiology report, CXR image, and intention visualization**

**Fig. 5:**
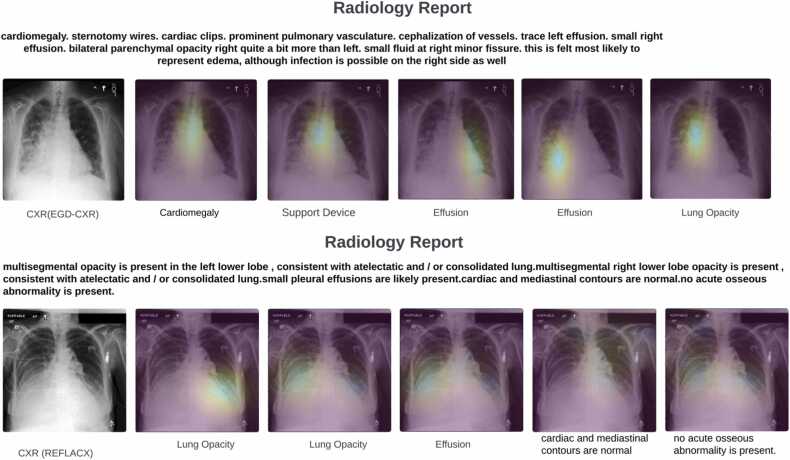
The intentions of radiologists and their corresponding regions of interest are illustrated. This figure depicts the specific areas within the image that radiologists focus on for each diagnosis mentioned in the report. One sample from both datasets is presented here.

Fig. 5 offers a demonstration of our system's ability to accurately predict radiologists’ intentions and highlight the corresponding ROI on CXR images, effectively simulating the diagnostic focus during image interpretation. The figure showcases two randomly selected images from the test sets of the EGD-CXR and REFLACX datasets, providing visual evidence of the system's proficiency in identifying multiple ROIs associated with a single abnormality. For instance, in the EGD-CXR example, the radiologist's report indicates the presence of effusion in both the left and right lungs and our system successfully pinpoints the relevant areas in both lungs on the CXR image. Similarly, in the REFLACX example, the system not only contextualizes labels generated by CheXpert but also identifies additional diagnostic features, highlighting its utility in providing comprehensive visual explanations that extend beyond automated labels. This capability is particularly valuable for student radiologists, as it aids in learning the basic anatomical structures and understanding the subtleties of diagnostic reasoning. The visualizations underscore the potential of our approach to enhance radiological training and improve the interpretability of AI-assisted diagnosis in clinical settings.

a. **Natural language generation metrics**

The model predicts text containing various intentions along with their corresponding time steps. Ultimately, we are using the Large Language model as an engine in the TGID module and the final output is in the form of text. It is always good to evaluate the generated text using NLG metrics. NLG metrics serve as a valuable tool in gauging the model's text generation proficiency. While NLG metrics may not be the optimal measure for evaluating predicted time steps for each intention, they provide a holistic indication that the model is generating meaningful output. Table 2 displays n-gram Blue scores and CIDEr scores [67] for various state-of-the-art methods, indicating that our model excels in generating intentions with accurate temporal grounding compared to the state-of-the-art DVC model PDVC [23]. We trained and tested the TGID module on the REFLACX and EGD-CXR datasets respectively. Table 2 shows the NLG metrics on the test set of EGD-CXR and REFLACX. As shown in Table 2, our proposed TGID module outperforms the state-of-the-art model in both the datasets EGD-CXR and REFLACX. TGID has a better CIDEr score on REFLACX compared to the EGD-CXR due to the larger size of the REFLACX dataset. This shows that the TGID model generalizes better when the dataset is large.

b. Evaluation of temporal groundings using time-based metric for intentions

**Table 2:** Comparison to the SoTA for Intention temporal grounding on two datasets ( REFLACX and EGD-CXR) recorded on different radiologists of varying experience. V/F/O refers to visual/flow/object features.

**Method**	**Dataset**	**Backbone**	**Blue-1**	**Blue-2**	**Blue-3**	**Blue-4**	**CIDEr**
PDVC[23]	EGD-CXR	V (CLIP)	0.19	0.17	0.12	0.10	1.7
PDVC[23]	REFLACX	V (CLIP)	0.20	0.19	0.14	0.13	1.9
**TGID (Our)**	**EGD-CXR**	**ResNet−101**	**0.44**	**0.44**	**0.43**	**0.43**	**3.5**
**TGID(Our)**	**REFLACX**	**ResNet−101**	**0.46**	**0.47**	**0.45**	**0.43**	**3.9**

**Fig. 6:**
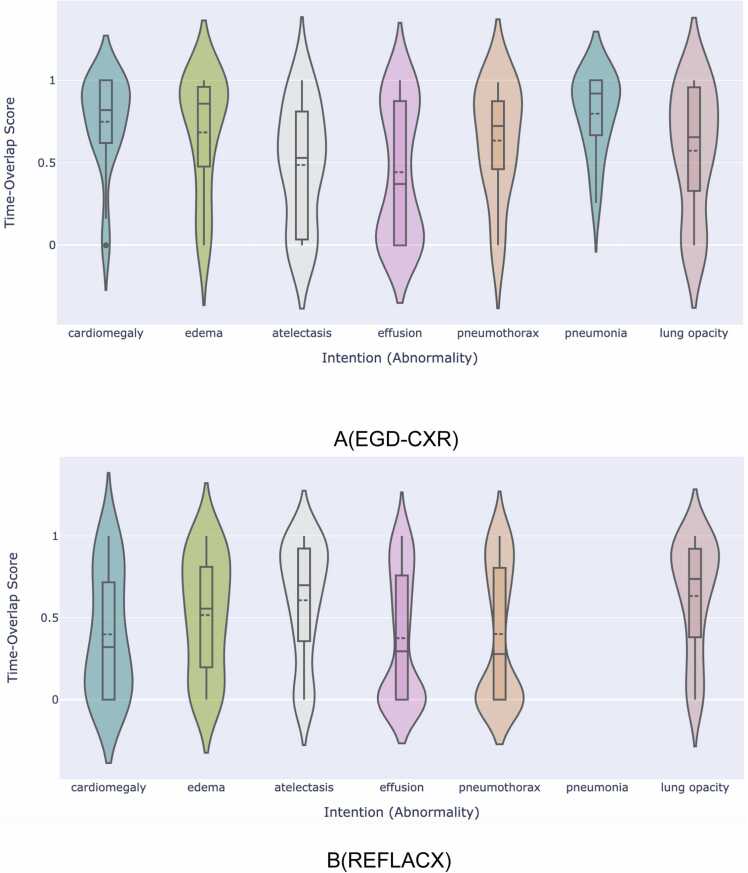
This figure illustrates the comprehensive performance of the TGID module across all predicted time steps in the intention sequence. It provides violin plots representing the distribution of time-overlap scores for different intentions ( abnormalities). It consists of two subfigures: Subfigure- A represents the TGID module’s performance on the EGD-CXR dataset and Subfigure- B represents the TGID module’s performance on the REFLACX dataset.

We conducted a comprehensive evaluation of the TGID module’s overall performance in predicting time steps for all abnormalities. Fig. 6 illustrates a violin plot showing the distribution of time-overlap scores for the predicted time steps of all intentions in the intention sequence for the EGD-CXR and REFLACX datasets separately. This visualization highlights the distribution of time-overlap scores for each disease, showcasing the TGID module's capability to accurately predict time steps and its ability to capture human cognitive processes.

Fig. 6(a) focuses on the time-overlap scores for the REFLACX dataset, which consists of eye gaze data recorded from multiple radiologists with varying levels of experience. The time-overlap scores for the REFLACX dataset are generally noisier compared to the EGD-CXR dataset due to this variation in experience, yet the model effectively handles this noise and still achieves strong predictions of time steps. A notable observation is the decreased time-overlap score for cardiomegaly in the REFLACX dataset. This is likely because different radiologists diagnose cardiomegaly using different visual cues where some may focus on the heart, while others may also consider the area around the lungs. As a result, this makes it challenging for the model to consistently identify the correct region for cardiomegaly in the REFLACX dataset.

Table 3 presents the mean and median overlap scores for various intentions across the EGD-CXR and REFLACX datasets, along with 95% confidence intervals (CI) based on 1000 bootstrapped samples. These scores assess the model’s accuracy in predicting temporal intervals associated with each disease, revealing performance variations influenced by dataset characteristics and intention types.

**Table 3:** Comparison of mean and median overlap scores of each intention ( Abnormality or Label) for EGD-CXR and REFLCAX datasets, along with 95% confidence intervals (CI).

**Disease**	**Mean-Overlap score ( EGD-CXR)** **With [95% CI (Upper, Lower)]**	**Median-Overlap score ( EGD-CXR)**	**Mean-Overlap score ( REFLCAX)** **With [95% CI (Upper, Lower)]**	**Median-Overlap score ( REFLCAX)**
Cardiomegaly	0.74(0.67,0.82)	0.81	0.39(0.28,0.52)	0.32
Edema	0.68(0.57,0.78)	0.85	0.51(0.42,0.59)	0.55
Pneumonia	0.79(0.67,0.91)	0.91	_	_
Atelectasis	0.48(0.36,0.61)	0.52	0.60(0.54,0.66)	0.69
Effusion	0.44(0.35,0.52)	0.37	0.37(0.33,0.41)	0.29
Pneumothorax	0.63(0.46,0.78)	0.72	0.40(0.35,0.44)	0.27
Lung Opacity	0.57(0.45,0.68)	0.65	0.63(0.56,0.69)	0.73

As shown in Table 3, the model's performance differs across intentions and datasets, with inter-observer variability among radiologists playing a significant role. A key factor contributing to this variability is the difference in radiologists' expertise levels [68]. For intentions such as cardiomegaly, edema, effusion, and pneumothorax, overlap scores drop significantly in the REFLACX dataset compared to EGD-CXR, likely due to noise introduced by differences in radiologist expertise. Effusion and pneumothorax, in particular, show consistently lower overlap scores across both datasets compared to other intentions due to their subtle, position-dependent presentations and overlap with other lung abnormalities. In contrast, pneumonia, evaluated only in EGD-CXR (due to its absence in REFLACX), demonstrates strong and consistent performance. Atelectasis remains stable across datasets, performing slightly better in REFLACX, while lung opacity is detected well in both datasets, particularly in REFLACX, likely due to its distinct appearance on imaging.

Table 3 further emphasizes that although the model performed well overall, accuracy is influenced by the dataset. Inter-observer variability, especially in REFLACX, contributes to discrepancies in temporal predictions. The model tends to perform better on datasets with more consistent diagnostic criteria and minimum inter-observer variability, such as EGD-CXR, highlighting the importance of considering both dataset characteristics and radiologist variability when evaluating model performance.

One challenge in evaluating the model's time-related predictions is that radiologists often use a non-linear approach when interpreting CXR, freely navigating the image rather than following a strict pattern. As a result, the TGID module may inaccurately predict the exact time step for a disease, leading to a 0 overlap score, even though the predicted region might still be correct. This discrepancy arises because radiologists may revisit the same area, as reflected in the time intervals of the ground truth. We explore this issue with an example in the next section on vision-related metrics.

While time-related metrics provide a general assessment of how well the model predicts timesteps compared to the ground truth, they do not fully capture the alignment of the predicted region with the ground truth. To offer a more detailed evaluation of the predicted regions, we will focus on visual metrics in the following section.

c. Evaluation of extracted ROI using Vision-Based metric for Intentions

As previously noted, relying solely on time-related metrics may not be the most accurate approach for assessing the model's ability to highlight the region of interest for the missing diagnosis. To address this, we employ a segmentation metric known as Intersection over Union (IoU) to calculate the overlap between the true and predicted regions.

**Fig. 7:**
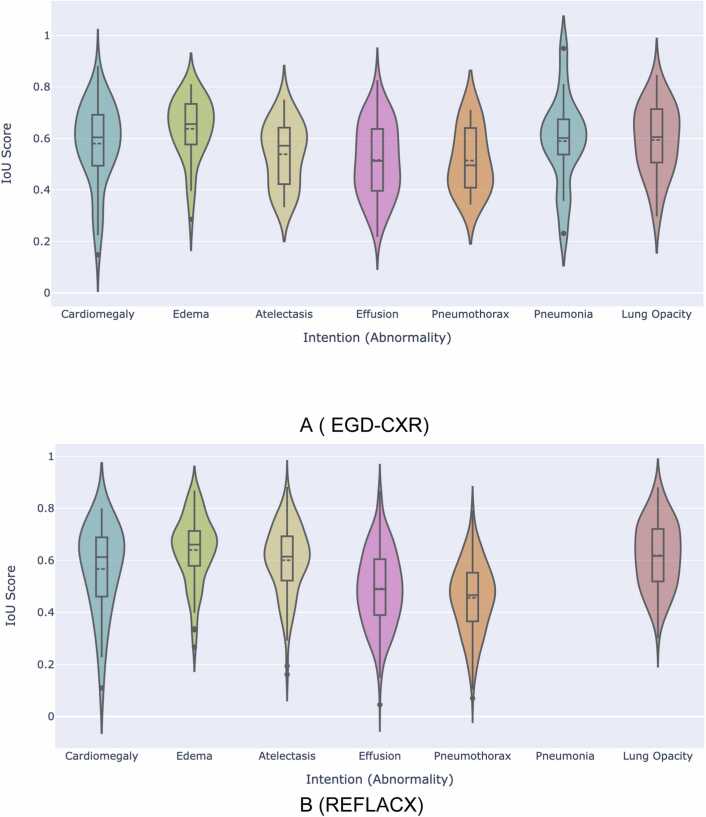
This figure illustrates the comprehensive performance of our proposed system across all predicted (ROI))regions of interest in the intention sequence. It provides a holistic view of the TGID module's effectiveness in identifying the region of interest for each intention(abnormality) in the report. It consists of two subfigures: Subfigure- A represents the system’s performance on the EGD-CXR dataset and Subfigure- B represents the TGID system’s performance on the REFLACX dataset.

In Fig. 7, we present a comprehensive distribution of the IoU scores for the entire diagnosis in our proposed system. It has two subfigures, Figs. 7(a) and 7(b). Fig. 7(a) represents the IoU scores for the REFLACX data and Fig. 7(b) shows the IoU scores of the EGD-CXR dataset. In the case of EGD-CXR, The IoU scores for all diseases showcase an almost symmetrical distribution around the mean, with closely aligned mean and median values for each disease. A comparison between Fig. 6(a), illustrating the distribution of overlap scores for each disease, and Fig. 7(a), which depicts the IoU score for each, reveals slight differences in distribution patterns. For instance, in the case of Edema, overlap scores may occasionally be zero, yet the model accurately predicts the correct region of interest. This divergence from the distribution pattern of IoU scores, which does not include zero for Edema, suggests that the predicted region of interest consistently corresponds to the ground truth. The same pattern holds for Pneumonia as well. This observation underscores the model's ability to effectively learn abnormal regions associated with each disease. The occurrence of zero overlap scores can be attributed to the radiologist’s tendency to employ a more efficient, free, and global search, rather than following a preconceived orderly pattern when scanning the CXR image [69]. As we can also see the IoU scores of the REFLACX dataset are a little noisy and follow the multimodal distribution. It is due to the previously defined fact that the REFLACX dataset consists of the data of multiple varying experienced radiologists which makes the learning a little noisy.

Additionally, we include a table presenting the mean and median IoU scores for each disease, offering insight into the TGID module's overall performance. We also calculate the 95% confidence interval using the bootstrap method for the mean-IoU score. The number of bootstrap samples is set to 1000. Table 4 showcases the module's effectiveness in identifying regions associated with different abnormalities for the EGD-CXR and REFLACX datasets.

**Table 4:** This table provides the mean and median Intersection over Union (IoU) scores for the predicted regions of interest corresponding to each intention(abnormality). It offers a comprehensive numerical assessment of the performance of the TGID module for individual abnormalities.

**Disease**	**Mean-IoU ( EGD-CXR)** **With [95% CI (Upper, Lower)]**	**Median-IoU ( EGD-CXR)**	**Mean-IoU ( REFLCAX)** **With [95% CI (Upper, Lower)]**	**Median-IoU ( REFLCAX)**
Cardiomegaly	0.58(0.53,0.62)	0.60	0.56(0.50,0.61)	0.61
Edema	0.63(0.59,0.67)	0.65	0.63(0.60,0.67)	0.66
Pneumonia	0.59(0.50,0.67)	0.60	_	_
Atelectasis	0.53(0.49,0.58)	0.51	0.60(0.57,0.62)	0.61
Pleural Effusion	0.51(0.48,0.54)	0.51	0.49(0.47,0.50)	0.48
Pneumothorax	0.51(0.45,0.57)	0.30	0.45(0.44,0.47)	0.46
Lung Opacity	0.59(0.54,0.63)	0.63	0.61(0.59,0.64)	0.61

Cardiomegaly, edema, and lung opacity show consistently strong IoU scores across both datasets, indicating the model's ability to reliably identify these intentions despite inter-observer variability. In contrast to the significant performance differences observed in overlap time scores, IoU performance remains stable. This suggests that while the model may struggle with pinpointing the exact timeframes for these intentions due to the non-linear diagnostic approach of radiologists, it still accurately identifies the relevant anatomical regions.

Pleural effusion and pneumothorax, despite showing lower performance in overlap time scores, also have relatively moderate IoU scores. This highlights that the model can detect the general region affected by these intentions, though variability in radiologist interpretation and the subtler presentation of these abnormalities make precise temporal detection difficult. Pneumonia, evaluated only in the EGD-CXR dataset, and atelectasis show stable IoU performance, with atelectasis performing better in REFLACX, possibly due to clearer imaging intentions.

The overall findings emphasize that IoU scores demonstrate the model's ability to locate the affected regions consistently, even when temporal prediction struggles due to radiologists' variability in how they interpret and navigate images.

A. Impact of Varying Radiologist Experience in REFLACX Dataset
  The REFLACX dataset includes eye gaze data recorded on multiple radiologists, each potentially bringing different interpretations and visual search patterns in the data, a phenomenon known as inter-observer variability [62]. This variability in radiologists' visual search patterns can lead to inconsistencies in the ground truth labels, making it difficult for the model to learn and generalize effectively. As shown in Table 4, Our proposed system shows a better IoU for most of the intentions in comparison to the EGD-CXR which shows the noise-resilient nature of our proposed system. The TGID module of our proposed system can deal with the noise introduced due to inter-observer variability. The lower median IoU scores were observed for Lung Opavity and Pleural Effusion in the REFLACX dataset compared to the EGD-CXR dataset. This could be attributed to the inconsistent localization of intentions or inter-observer variability, which introduces noise and reduces the model’s performance for these two abnormalities.
B. Discussion on Mean vs. Median IoU

The differences between the mean and median IoU values provide insights into the distribution and variability of the model's performance. The mean IoU represents the average performance across all cases but can be skewed by extreme values (either very high or very low IoU scores). In contrast, the median IoU represents the middle value when all cases are ordered, providing a better measure of the typical case performance.

For instance, in the case of pneumothorax, the mean IoU in the EGD-CXR dataset (0.51) is significantly higher than the median (0.30), indicating that there are a few cases with relatively high IoU scores that raise the average, while the majority of cases have lower scores. This discrepancy suggests that the model struggles with consistent performance across all pneumothorax cases, potentially due to the small and sharply defined nature of the pneumothorax regions.

Conversely, intentions like Edema and Lung Opacity have mean and median IoUs that are quite close, suggesting a more uniform performance across different cases. This implies that the model has a relatively steady ability to detect these intentions, likely due to their more diffuse or consistently presented nature in the datasets.

D. **Visual comparison: Predicted vs. ground truth intention regions**

**Fig. 8:**
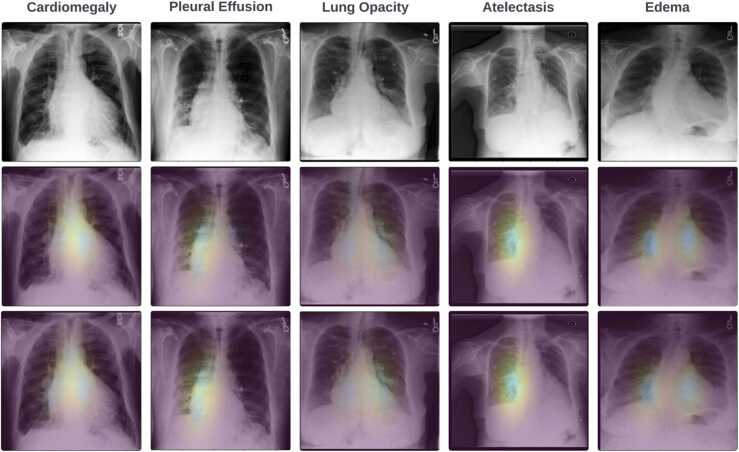
Visual comparison of predicted and ground truth ROI for different intentions. The first row features the original CXR image, the second row displays the ground truth image highlighting the intention region, and the third row presents the predicted intention region generated by the TGID module.

Fig. 8 presents a comparative analysis of the predicted and ground truth regions of interest for various intentions (abnormalities). As previously described, the TGID module predicts the time intervals (start and end) for each intention. Subsequently, the Region Extraction (RE) module utilizes these time intervals to extract frames from the fixation video. It then calculates a mean frame by consolidating multiple frames into a single image that accurately represents the region of interest for each specific intention. The results for five intentions are displayed, demonstrating the model's effectiveness in predicting abnormality regions. Notably, our proposed system can identify multiple regions associated with a specific abnormality mentioned by the radiologist in their report. For example, in the case of lung opacity, our model accurately predicts the opacity in both lung bases.

## 7 Conclusion

Our work presents a pioneer approach to aligning AI outputs with radiologist’s cognitive processes through the innovative use of LMM. By integrating TGID and RE, the proposed system effectively bridges the gap between human cognition and AI interpretation in medical imaging. The detailed evaluation, leveraging both natural language generation metrics and vision-based metrics, underscores the system's robust performance in predicting temporal intentions and accurately identifying regions of interest. Despite challenges arising from dataset variability and radiologist experience, our approach demonstrates significant improvements in diagnostic accuracy and interpretability. This advancement holds promise for enhancing radiological practice, supporting ongoing learning, and fostering more effective communication between experienced radiologists and trainees. In future work, we want to extend our system for the CT and MRI scans since they are quite complex and it will be very useful to reverse engineer the complex cognitive processes of radiologists on the CT and MRI.

## Author statement

This research was conducted by a multidisciplinary team with expertise in electrical and computer engineering, computer science, and thoracic imaging, providing a well-rounded and comprehensive approach. The project was led by Akash Awasthi, a PhD student in the Department of Electrical and Computer Engineering at the University of Houston, under the mentorship of Dr. Hien Van Nguyen, Associate Professor at the same institution. Dr. Zhigang Deng, Moores Professor of Computer Science, and Dr. Ngan Le, Assistant Professor of Computer Science & Computer Engineering at the University of Arkansas, offered valuable technical expertise. Clinical insights were provided by Dr. Carol C. Wu, Professor of Thoracic Imaging at The University of Texas MD Anderson Cancer Center, and Dr. Rishi Agrawal, Associate Professor in the same department, ensuring the clinical relevance and applicability of the study.

## CRediT authorship contribution statement

**Hien Van Nguyen:** Writing – review & editing, Writing – original draft, Supervision, Resources, Project administration, Funding acquisition. **Rishi Agrawal:** Supervision. **Carol wu:** Supervision. **Ngan Le:** Supervision. **zhigang Deng:** Supervision. **Awasthi Akash:** Writing – review & editing, Writing – original draft, Visualization, Validation, Software, Resources, Methodology, Investigation, Formal analysis, Data curation, Conceptualization.

## Declaration of Competing Interest

All authors declare that there are no conflicts of interest related to this work.
