# Supplementary material for: Bridging human and machine intelligence: Reverse-engineering radiologist intentions for clinical trust and adoption
Source: Comput Struct Biotechnol J. 2024 Nov 8;24:711–23. doi: 10.1016/j.csbj.2024.11.012 (PMC11629193; doi:10.1016/j.csbj.2024.11.012)

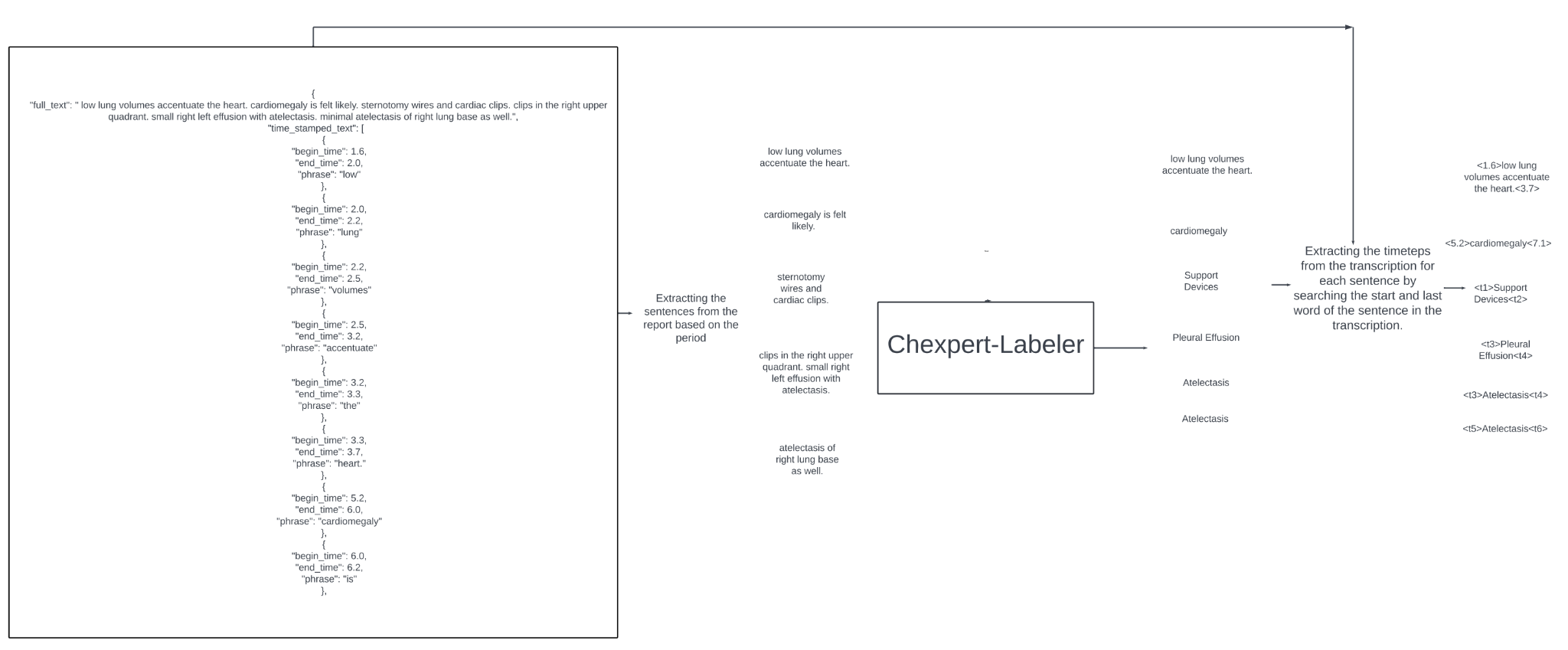


Figure 1: Overview of the Report Summarization and Timesetp extraction process used in creating the ground-truth file for intention detection and localization.

We also show some of the example region of interest predictions corresponding to its expert annotations available in REFLACX data for some abnormalities. The robustness of our system lies in its ability to detect meaningful signals in the data, making it resilient to the noise introduced by temporal misalignment.

**Proposed system Prediction( Comparison of the ROI predicted by the proposed system vs Expert Annotations )**

Edema Pleural abnormality Cardiac silhouette is mildly enlarged.


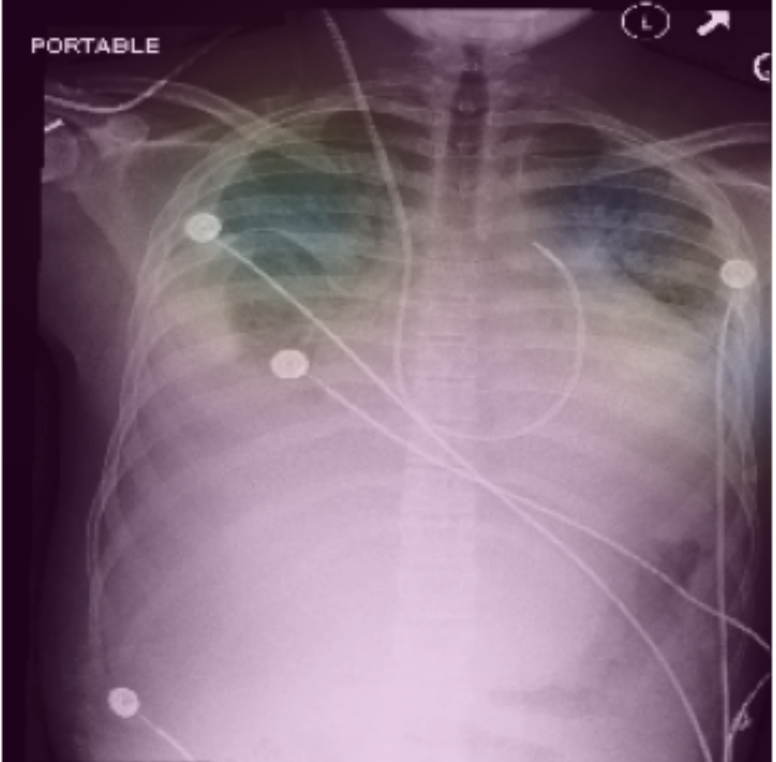

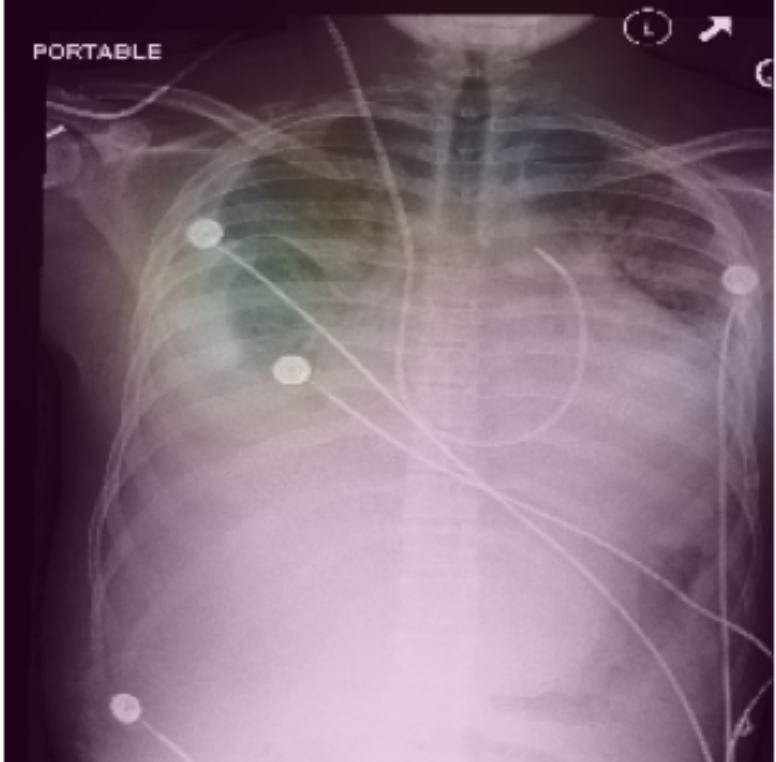

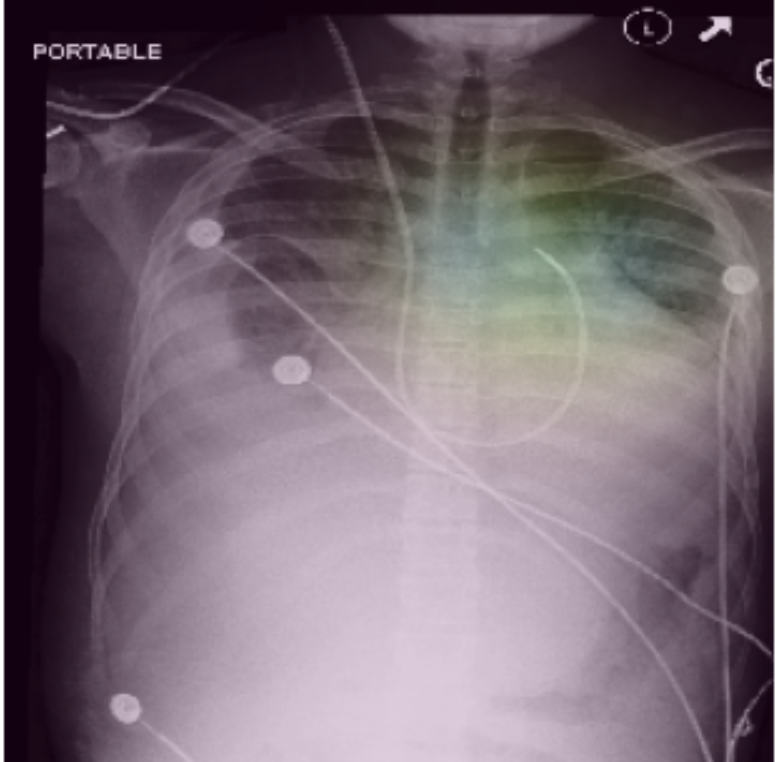

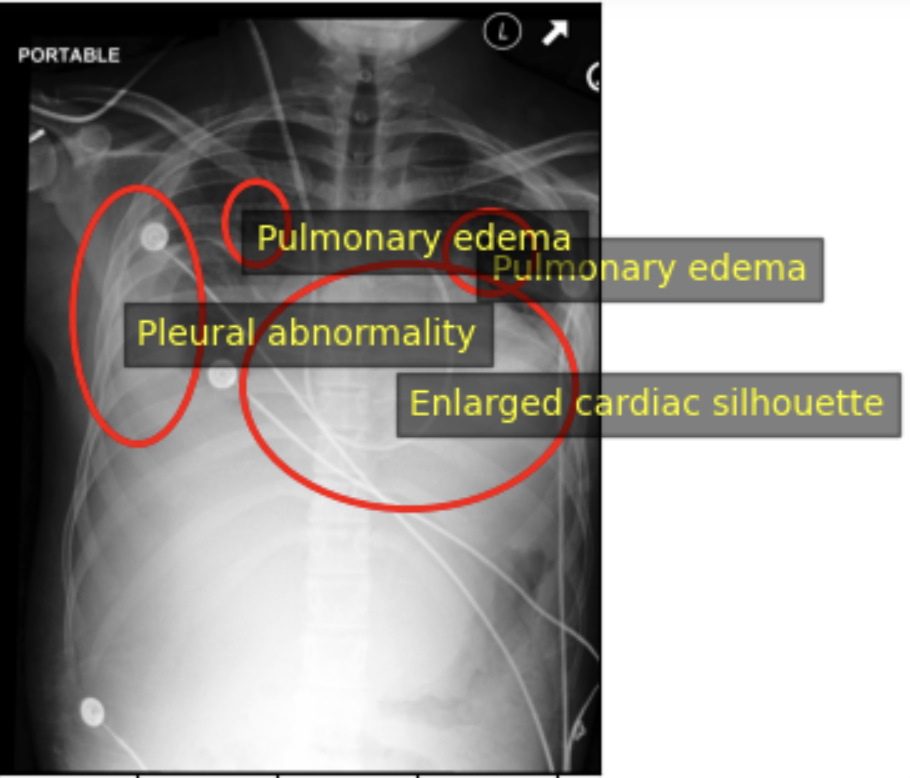


Expert Annotation

Consolidation Expert Annotation


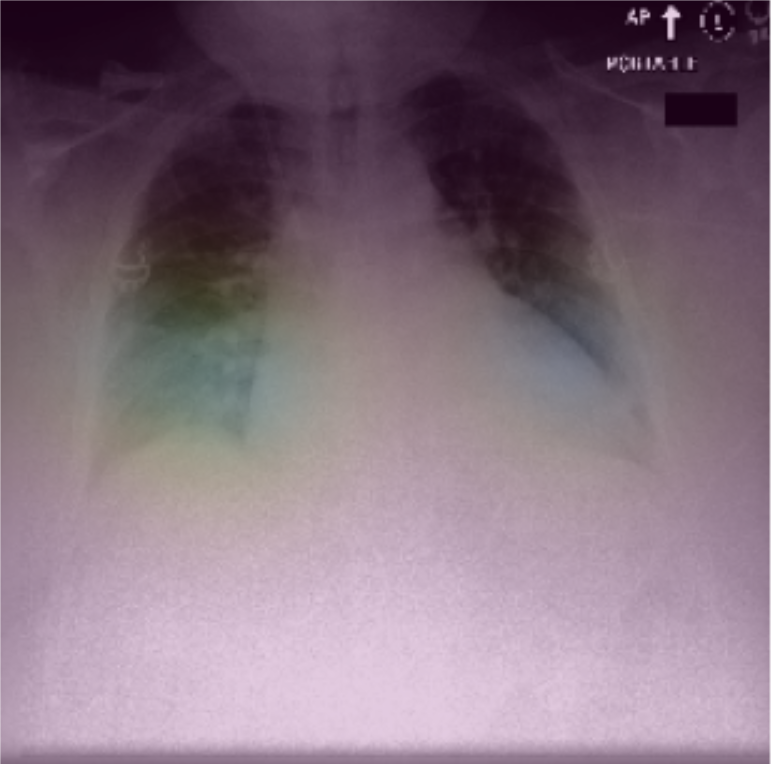

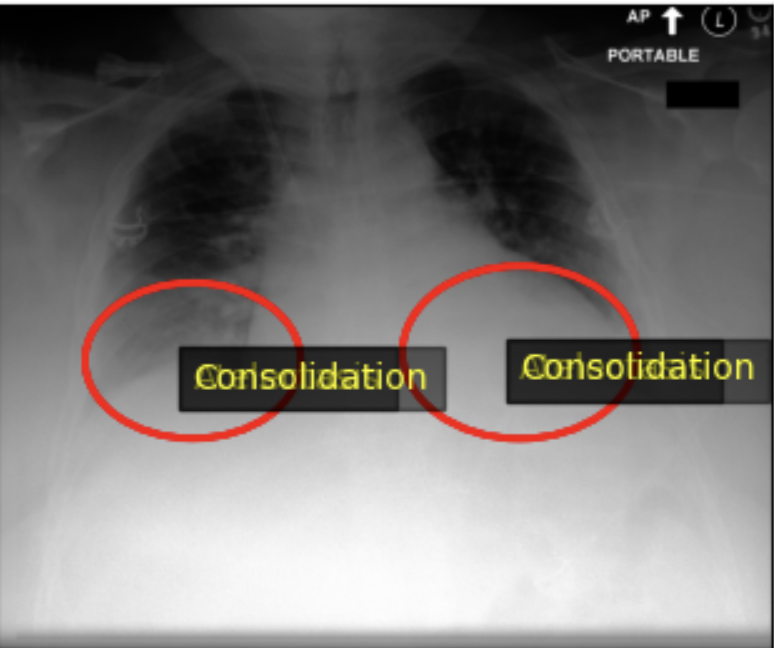


Lung Opacity Expert Annotation


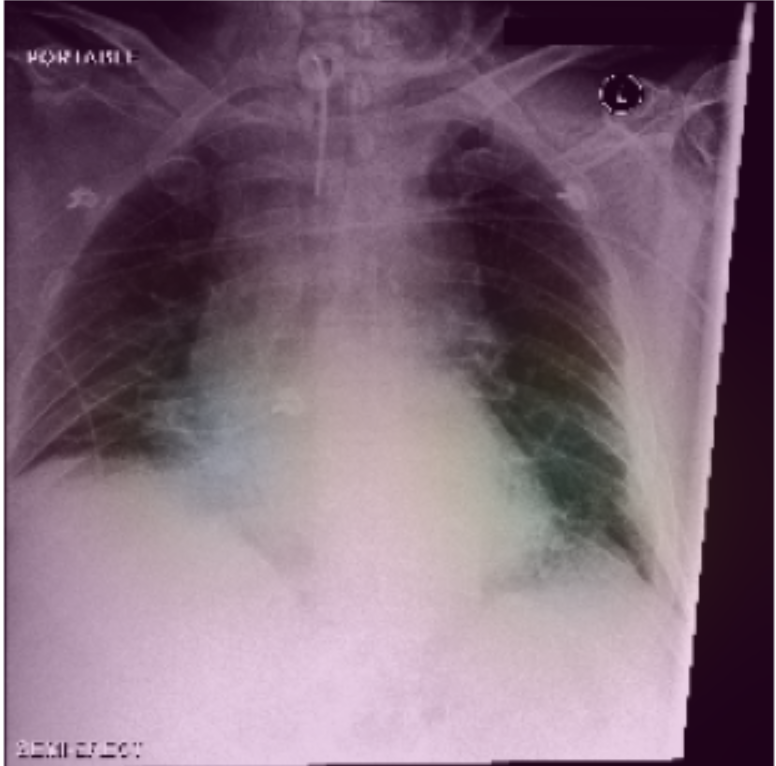

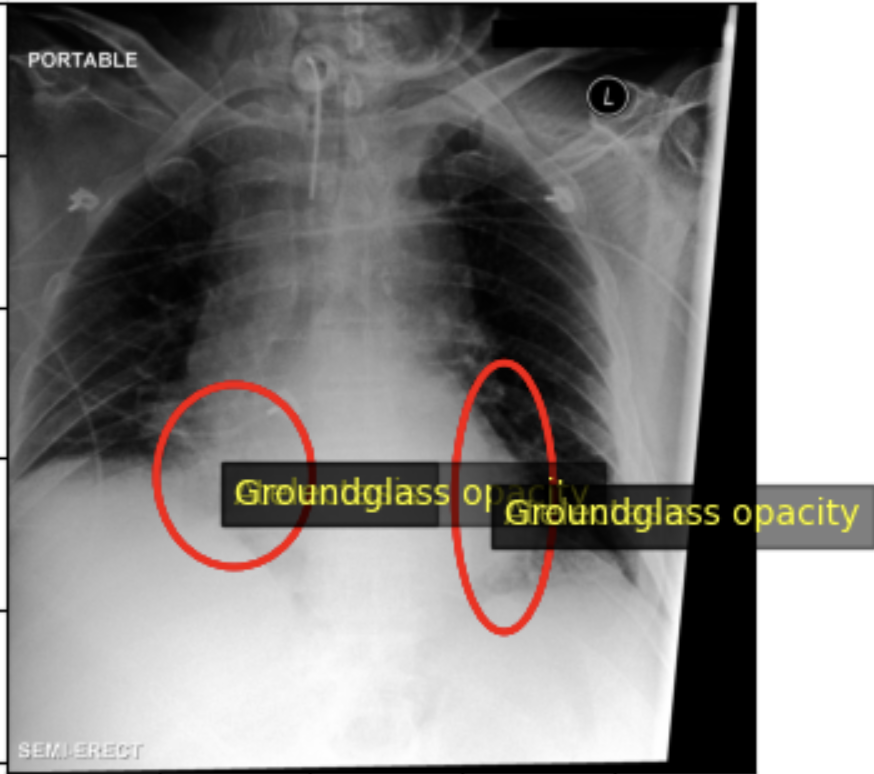


Pleural thickening is present in the basal Expert Annotation

right hemithorax.


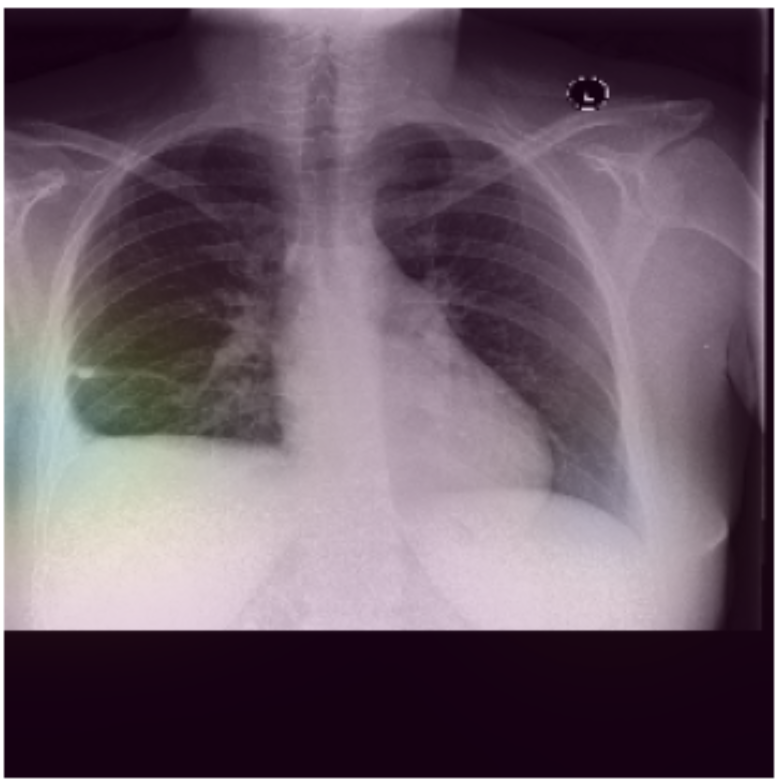

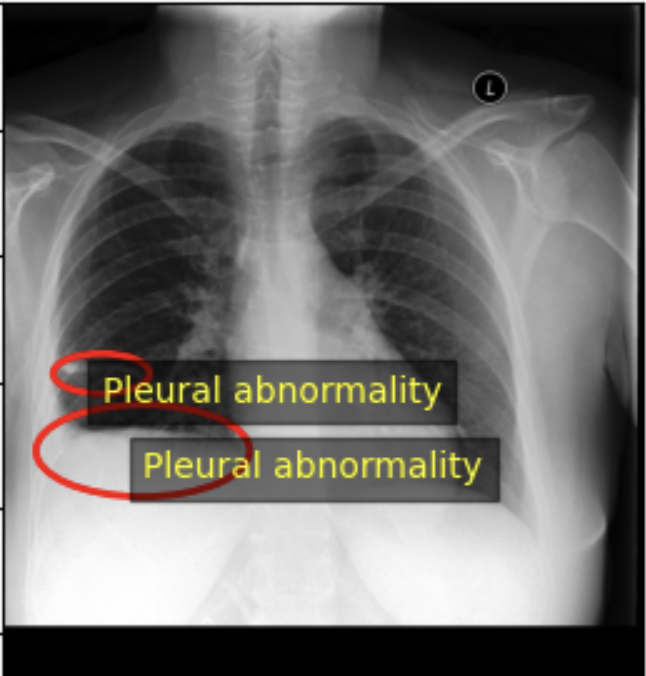


Lung Opacity Expert Annotation


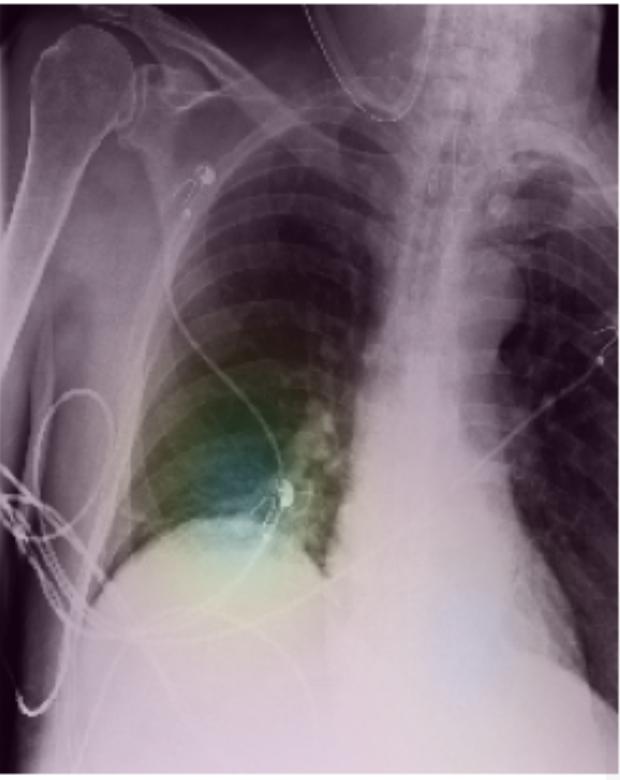

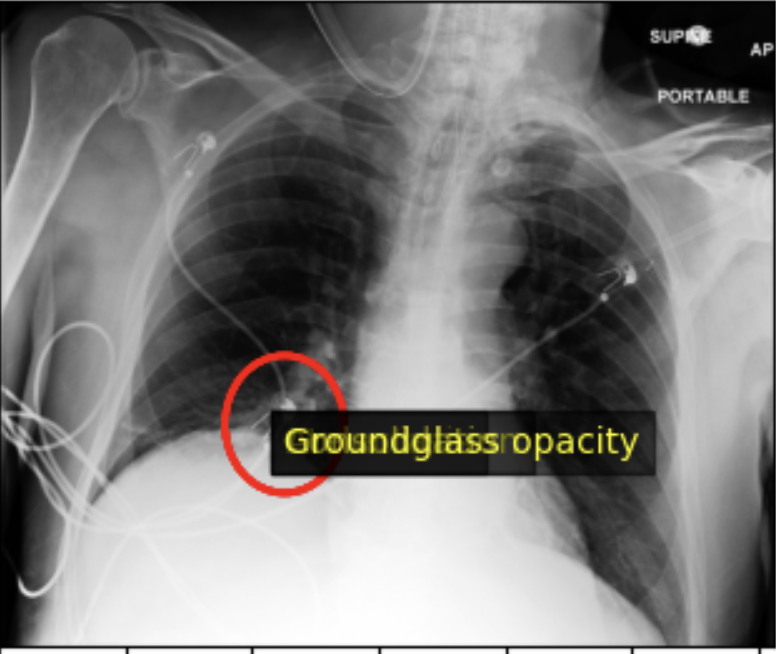

Supplement: Supplementary file 1 — Supplementary material [file mmc1.docx]
